# Upregulation of excitatory neurons and downregulation of inhibitory neurons in barrel cortex are associated with loss of whisker inputs

**DOI:** 10.1186/1756-6606-6-2

**Published:** 2013-01-03

**Authors:** Guanjun Zhang, Zilong Gao, Sudong Guan, Yan Zhu, Jin-Hui Wang

**Affiliations:** 1 Department of Physiology, Bengbu Medical College, Bengbu, Anhui Province 233000, China; 2 State Key Lab of Brain and Cognitive Science, Institute of Biophysics, Chinese Academy of Sciences, Beijing 100101, China; 3 University of Chinese Academy of Sciences, Beijing 100049, China

**Keywords:** Neural plasticity, Neuron, Synapse, GABA, Glutamate, Barrel cortex and whisker

## Abstract

Loss of a sensory input causes the hypersensitivity in other modalities. In addition to cross-modal plasticity, the sensory cortices without receiving inputs undergo the plastic changes. It is not clear how the different types of neurons and synapses in the sensory cortex coordinately change after input deficits in order to prevent loss of their functions and to be used for other modalities. We studied this subject in the barrel cortices from whiskers-trimmed mice vs. controls. After whisker trimming for a week, the intrinsic properties of pyramidal neurons and the transmission of excitatory synapses were upregulated in the barrel cortex, but inhibitory neurons and GABAergic synapses were downregulated. The morphological analyses indicated that the number of processes and spines in pyramidal neurons increased, whereas the processes of GABAergic neurons decreased in the barrel cortex. The upregulation of excitatory neurons and the downregulation of inhibitory neurons boost the activity of network neurons in the barrel cortex to be high levels, which prevent the loss of their functions and enhances their sensitivity to sensory inputs. These changes may prepare for attracting the innervations from sensory cortices and/or peripheral nerves for other modalities during cross-modal plasticity.

## Introduction

Behavioral experiences modify neuronal function and rewire neuronal circuits to change the brain structure and function, i.e., experience-dependent neural plasticity [1-8]. Despite its critical importance in developmental period [9], the experience-dependent neural plasticity may occur in the adulthood after removing the stabilized processes and shifting the excitation-inhibition balance [10-15]. The experience-dependent neuronal plasticity is believed to play important roles in the memory formation [16-21] and the behavioral rehabilitation [3,6]. In terms of the molecular mechanism, long-lasting neuronal activities in various experiences triggers the cellular nuclei to transcript certain genes and the cytoplasm to express the proteins relevant to the plasticity at the neurons and synapses through the diversified arrays [7,22,23]. How the different types of the neurons and synapses rewire their connections and reset their functions, i.e., cell-specific changes in the experience-dependent neural plasticity, remains an open question to be studied [24].

In terms of the cellular mechanism underlying experience-dependent neural plasticity, the model of whisker experiences has been used without organ injury. In these studies, trimming whiskers led to the following changes in the barrel cortex, such as alternations in dynamics of excitatory synapses [25,26], pathway-specific synaptic plasticity [27-30], dendritic reorganizations [31,32], new spine generation on dendrites [33,34], zinc-containing neural circuit reorganization [35,36], and downregulation in cortical responses [37,38]. These results indicate the crucial roles of synaptic plasticity and circuit rewire in experience-dependent neural plasticity. It remains unclear how the intrinsic properties at the different types of neurons, the signal transmission at the synapses and the morphology of their subcellular compartments in the barrel cortices are coordinately regulated in response to changing sensory experience.

We have investigated this subject in the barrel cortices from whisker-trimmed mice and controls, in which pyramidal neurons were genetically labeled by yellow fluorescent protein and GABAergic cells were labeled by green one. We analyzed the capability of these neurons to convert excitatory inputs into digital spikes and the intrinsic properties mediated by voltage-gated sodium channels (VGSC). We also analyzed the transmissions of glutamatergic and GABAergic synapses. In terms of their morphology, we analyzed their dendritic structure and spines. Our results indicate that the differentiated regulations in the excitatory and inhibitory units as well as the coordinated change in cellular function and morphology are associated with loss of whisker inputs.

## Results

**Figure 1:**
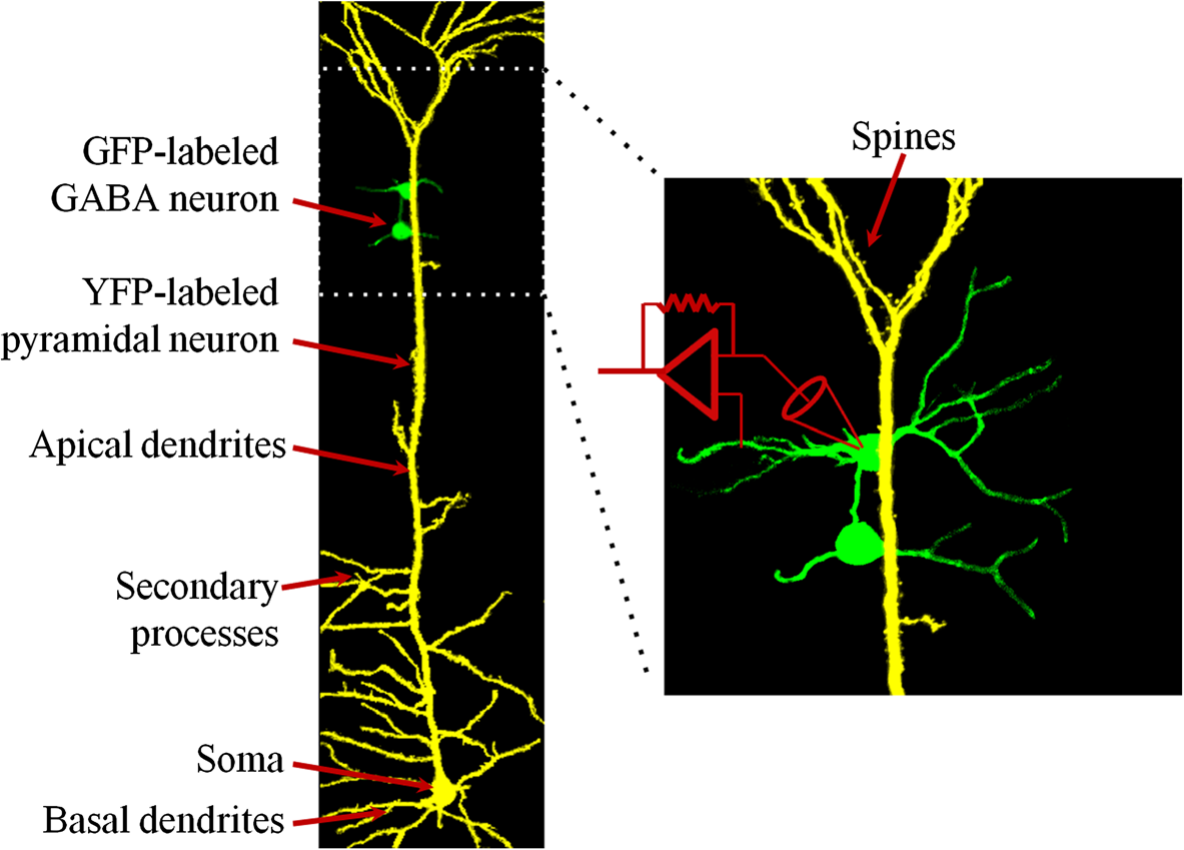
**Whole-cell recording on barrel cortical pyramidal and GABAergic neurons that are genetically labeled by yellow and green fluorescent proteins, respectively. **The morphological analyses are conducted for the processes and spines of apical and basal dendrites on pyramidal neurons as well as the processes on GABAergic neurons. Primary processes are those sprouted from somata, and secondary processes are those from the primary processes.

In studying the roles of barrel cortical excitatory and inhibitory neurons in experience-dependent neural plasticity, we divided mice into the groups of control and whisker trimming, whose whiskers were either intact or completely trimmed on the right side. The neurons were genetically labeled by fluorescent proteins, yellow for pyramidal cells and green for GABAergic cells (Figure 1). Active intrinsic properties were evaluated by spiking ability and threshold potentials. The ability of these neurons to receive inputs was estimated by analyzing synaptic transmission and their spines. The synapse functions were recorded by spontaneous postsynaptic currents on pyramidal neurons. Processes and spines were accounted under laser scanning confocal microscope. It is noteworthy that these functional and morphological studies were conducted on the left side of barrel cortices since whiskers were trimmed on the right side.

### The excitatory units in the barrel cortex are upregulated after loss of whisker inputs

**Figure 2:**
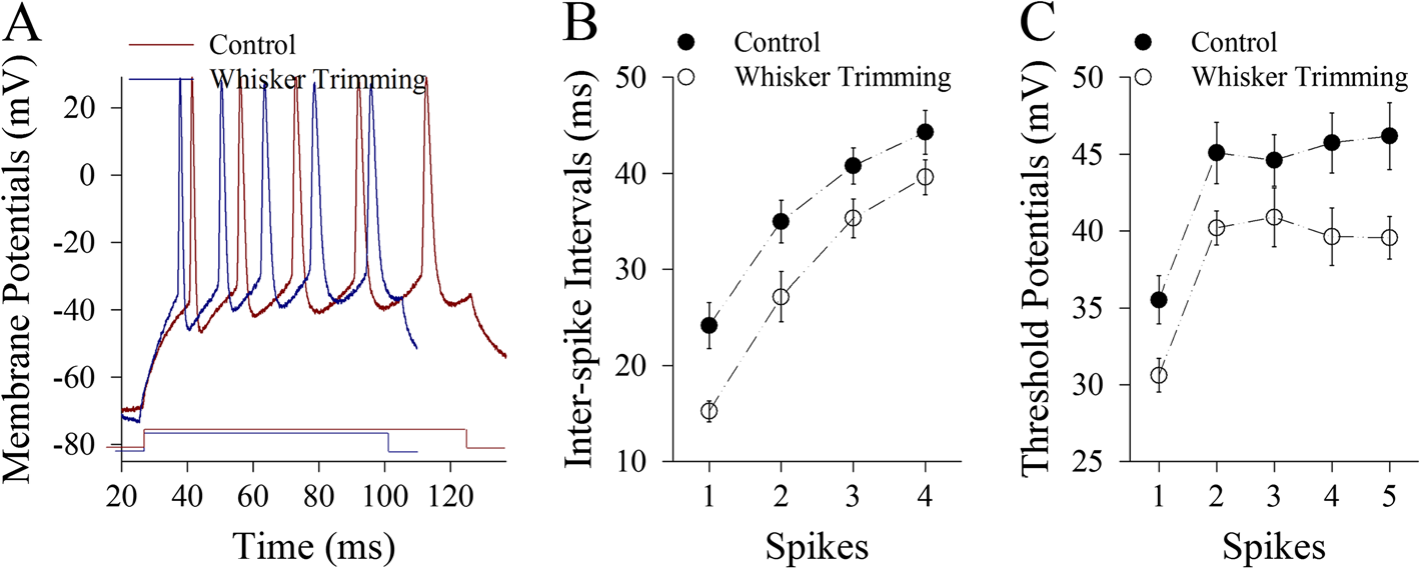
**The loss of whisker inputs upregulates the capability to produce action potentials in pyramidal neurons of the barrel cortex. **Sequential spikes were induced by depolarization pulses in an intensity that was a threshold for inducing a spike by 10 ms of pulse for each neuron. **A) **illustrates sequential spikes induced at pyramidal neurons from whisker-trimmed mouse (dark-blue trace) and control (dark-red one). **B)** illustrates inter-spike intervals for spike 1~2 to 4~5 in pyramidal neurons from whisker-trimmed mice (open symbols; n=15) and controls (filled; n=16, p<0.01). **C)** illustrates threshold potentials for spike 1~5 in pyramidal cells from whisker-trimmed mice (open symbols; n=15) and controls (filled; n=16, p<0.01).

Excitatory units in our study are pyramidal neurons and excitatory synapses in the barrel cortices. Their functions and processes/spines’ morphology are analyzed. In terms of the active intrinsic properties, Figure 2 illustrates the ability to convert excitatory inputs into digital spikes measured by inter-spike interval (ISI). These neurons after loss of whisker inputs appear to have higher ability to encode spikes (dark-blue trace in Figure 2A), compared with controls (dark-red). Figure 2B shows ISIs in pyramidal cells from whisker trimming (WT, open symbols) and control mice (filled). ISI values for spikes 1~2 up to 4~5 are 15.21±1.1, 27.16±2.6, 35.32±2 and 39.61±1.8 in the WT neurons (n=15); and 24.15±2.4, 34.9±2.2, 40.78±1.9 and 44.3±2.3 in the controls (n=16). ISI values for corresponding spikes in these two sources of neurons are statistically different (p<0.01). Therefore, the loss of whisker inputs enhances the capability of pyramidal neurons to convert excitatory inputs into digital spikes.

Figure 2C illustrates VGSC-mediated threshold potentials (Vts) at pyramidal neurons. Vts values for spikes 1 to 5 are 30.52±1.56, 40.19±1.1, 40.9±1.9, 39.62±1.88 and 39.56±1.39 in the WT neurons (open symbols; n=15), and are 35.52±1.56, 45.1±2, 44.61±1.68, 45.75±1.97 and 46.19±2.19 in the controls (filled, n=16). Vts values for corresponding spikes are significantly lower in the WT neurons than in the controls (p<0.01). Thus, a loss of whisker inputs reduces the threshold for firing spikes at barrel cortical pyramidal neurons.

**Figure 3:**
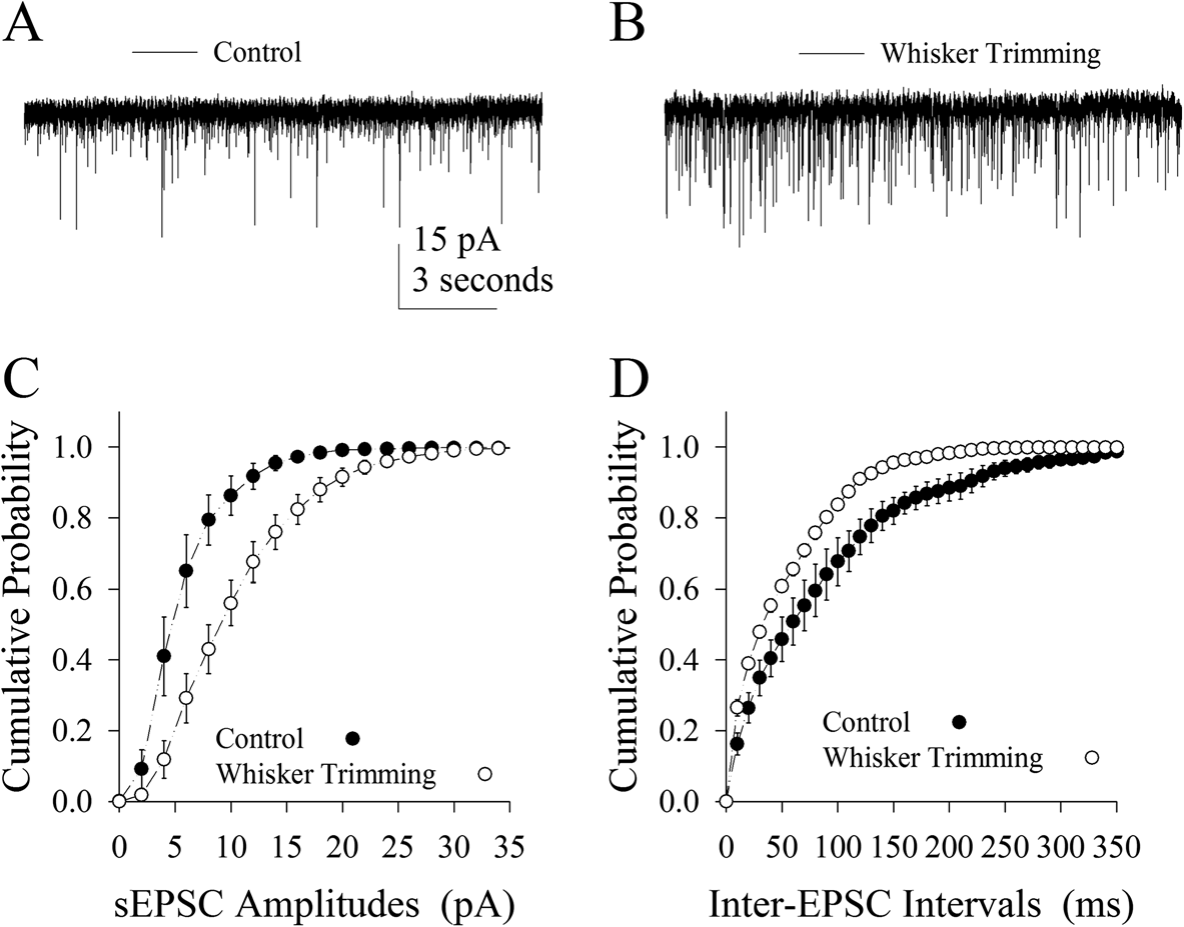
**A loss of whisker input upregulates the signal transmission of excitatory synapses on pyramidal neurons of the barrel cortex. **The strength of excitatory synapses was evaluated by recording spontaneous excitatory postsynaptic currents (sEPSC) in presence of 10 μM bicuculline, which were blocked by 10 μM CNQX and 40 μM D-AP5. **A) **illustrates sEPSCs recorded on a pyramidal neuron in the barrel cortex of a control mouse. **B) **illustrates sEPSCs recorded on a pyramidal neuron in the barrel cortex from a whisker-trimmed mouse. Calibration bars are 15 pA/3 seconds **C)** illustrates cumulative probability versus sEPSC amplitudes in barrel cortical pyramidal neurons from whisker-trimmed mice (open symbols, n=11) and controls (filled, n=12). **D)** illustrates cumulative probability versus inter-EPSC intervals in barrel cortical pyramidal neurons from whisker-trimmed mice (open symbols, n=11) and controls (filled, n=12).

Excitatory synaptic transmission was estimated by recording spontaneous excitatory postsynaptic currents (sEPSC) on pyramidal neurons. Figure 3 illustrates the effect of loss of whisker inputs on excitatory synaptic transmission. sEPSCs after whisker trimming appear higher (Figure 3B), compared to controls (Figure 3A). Figure 3C shows cumulative probability vs. sEPSC amplitudes from the WT neurons (open symbols, n=11) and the controls (filled, n=12). Figure 3D shows cumulative probability vs. inter-sEPSC intervals from the WT neurons (open symbols, n=11) and the controls (filled, n=12). Statistical analysis indicates that sEPSC amplitudes and frequencies (1/inter-sEPSC interval) are higher in the WT neurons than in the controls. Thus, the loss of whisker inputs enhances excitatory synaptic transmission including glutamate release probability and receptor responsiveness on the pyramidal neurons of the barrel cortices.

**Figure 4:**
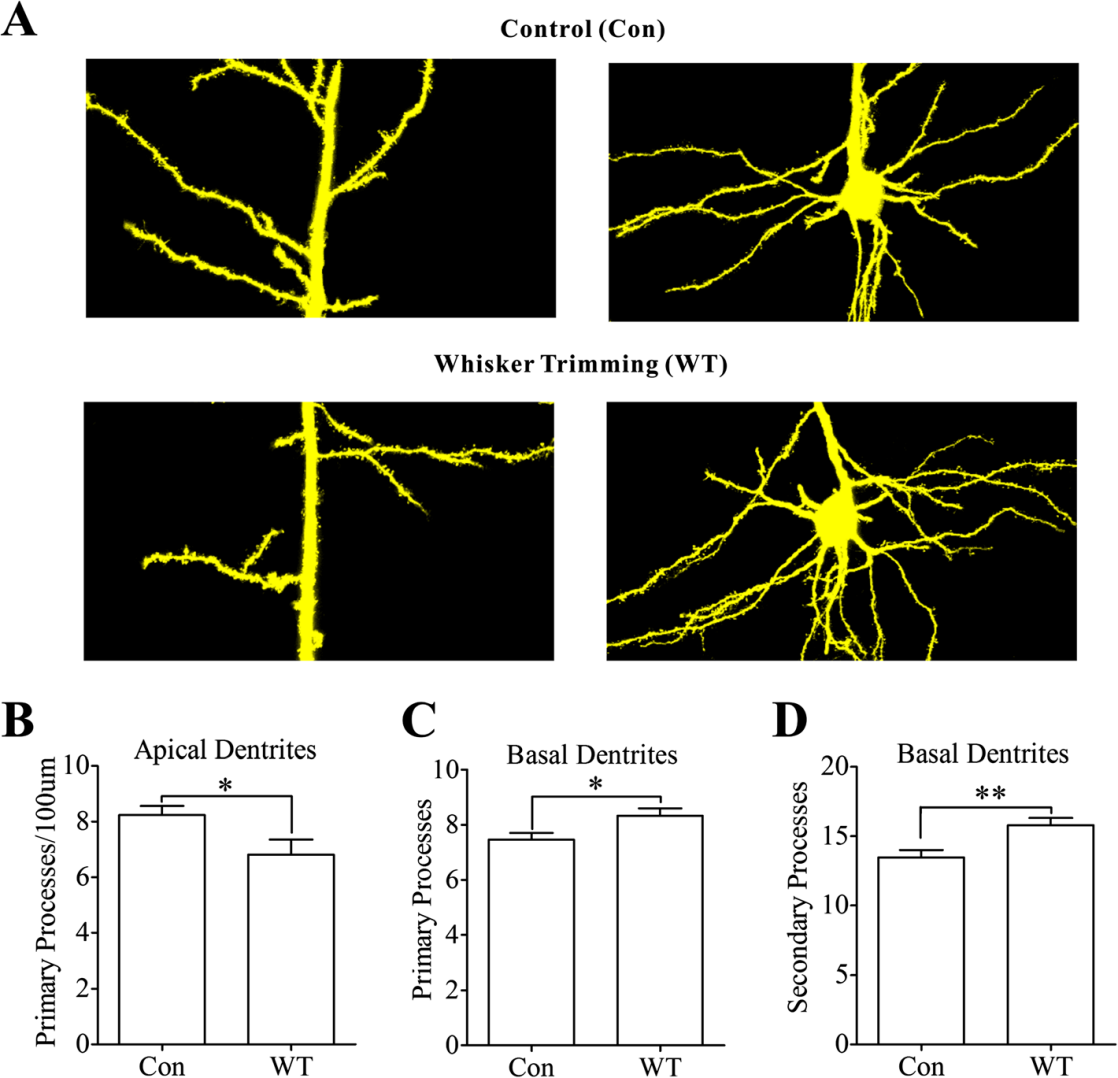
**A loss of whisker inputs leads to plastic changes in the apical and basal dendrites of pyramidal neurons in the barrel cortices. A) **Top panels show the images of apical dendrites (left) and basal dendrites (right) on pyramidal cells from mice without whisker trimming, i.e., control (Con). Bottom panels show the images of apical dendrites (left) and basal ones (right) on pyramidal neurons from mice with whisker trimming (WT). **B) **Statistical analysis illustrates primary processes per 100 μm on apical dendrites from whisker-trimmed mice and controls (n=18, p<0.05). **C)** illustrates primary processes on basal dendrites from whisker-trimmed mice and control (n=18, p<0.05). **D) **shows secondary processes on basal dendrites from whisker-trimmed mice and controls (n=18, p<0.01).

In terms of morphological changes of barrel cortical pyramidal neurons, we analyzed the densities of processes and spines, which reflected their capacity to receive the excitatory inputs. The processes and spines on pyramidal neurons were accounted from the images photographed by confocal microscope. Figure 4 shows the processes on apical and basal dendrites. The number of processes appears to change on the dendrites of pyramidal neurons from whisker-trimmed mice (bottom panels in Figure 4A), compared with those from controls (tops in Figure 4A). Statistical analyses in Figure 4B~D illustrate the number of processes on apical dendrites, primary and secondary processes on somata (basal dendrites) from the WT mice and the controls. The processes per 100 μm on apical dendrites are 6.8±0.54 in the WT neurons and 8.24±0.33 in the controls (Figure 4B; p<0.05, n=18). The primary processes of basal dendrites are 8.33±0.27 in the WT neurons and 7.46±0.24 in the controls (Figure 4C; p<0.05, n=18). The secondary processes from the basal dendrites are 15.78±0.54 in the WT neurons and 13.46±0.53 in the controls (Figure 4D; p<0.01, n=18). Thus, the area to receive synaptic inputs increases on the basal dendrites of pyramidal neurons, but decreases on their apical dendrites, after loss of whisker inputs.

**Figure 5:**
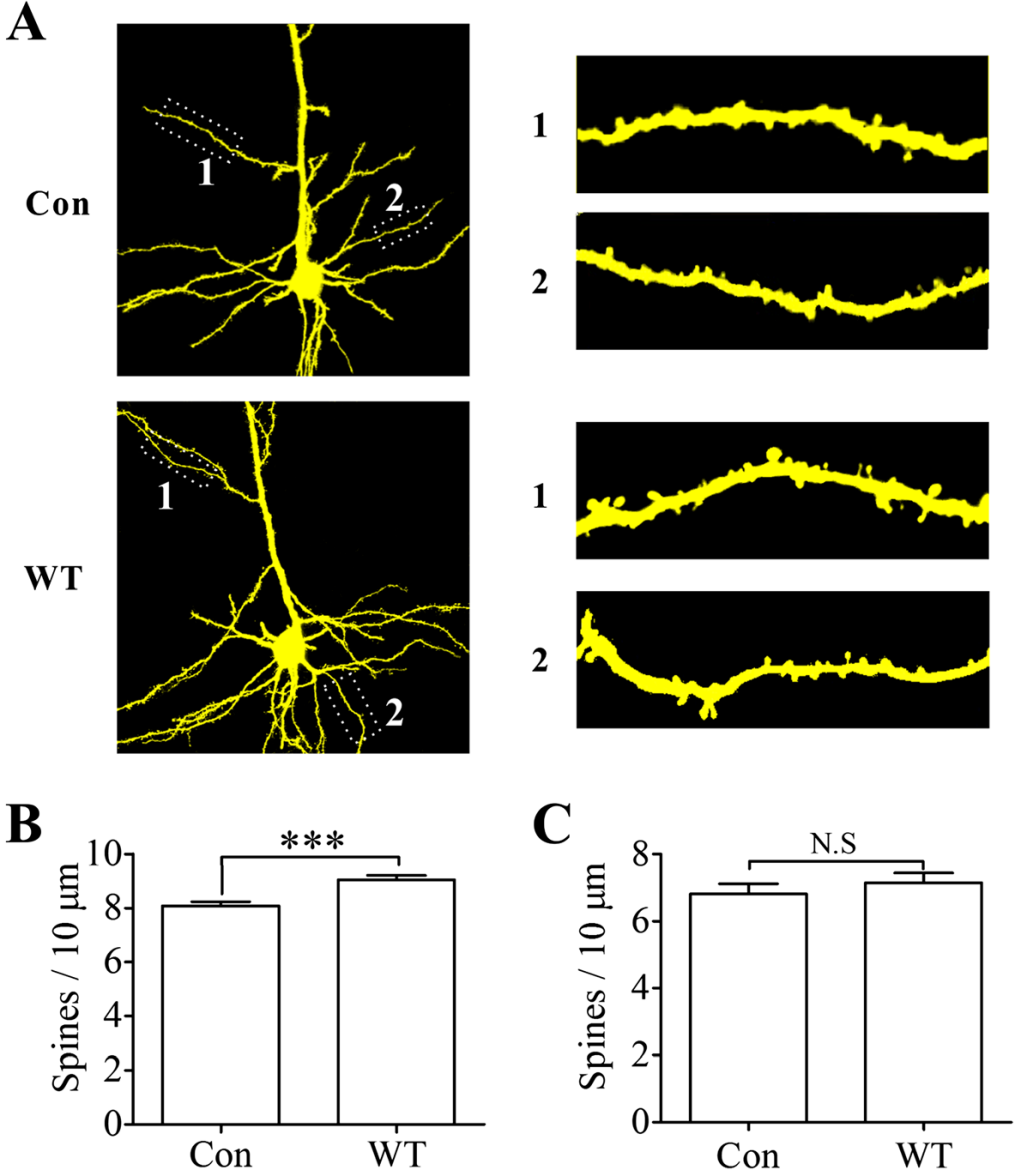
**A loss of whisker inputs leads to plastic changes in the spines of pyramidal neurons in the barrel cortices. A) **Top panels show the images of dendrites (left) and spines (right) on pyramidal neurons from mice of controls (Con). Bottom panels show the images of dendrites (left) and spines (right) on pyramidal neurons from mice with whisker trimming (WT). **B) **Statistical analysis shows spines per 10 μm on apical dendrites from whisker-trimmed mice and controls (n=13, p<0.001). **C) **shows spines on basal dendrites from whisker-trimmed mice and controls (n=12, p=0.35).

Figure 5 illustrates the density of spines on apical and basal dendrites. The number of spines appears to increase on the dendrites of pyramidal neurons from the WT mice (bottom panels in Figure 5A), compared to those from the controls (tops in Figure 5A). Statistical analyses in Figure 5B-C show the number of spines on the apical and basal dendrites of pyramidal cells from the WT mice and the controls. Spines per 10 μm on apical dendrites are 9.1±0.16 in the WT neurons and 8.1±0.15 in the controls (p<0.001, n=13). Spines per 10 μm on basal dendrites are 7.15±0.29 in the WT neurons and 6.81±0.31 in the controls (p=0.35, n=12). Therefore, the sites for receiving excitatory presynaptic inputs increase on the apical dendrites of pyramidal neurons after loss of whisker inputs.

In summary, loss of whisker inputs upregulates the functions of excitatory neurons and synapses as well as the areas and sites of receiving synaptic inputs in the barrel cortices. We subsequently studied the influence of loss of whisker inputs on the GABAergic inhibitory neurons and synapses in the barrel cortices.

### The inhibitory units in the barrel cortex are downregulated after loss of whisker inputs

Inhibitory units in our study included GABAergic neurons and their output synapses in the barrel cortices. Inter-spike intervals and threshold potential were analyzed to indicate active intrinsic properties. Inhibitory synaptic transmission was evaluated by recording spontaneous inhibitory postsynaptic currents (sIPSC) on pyramidal neurons. The processes on GABAergic neurons were accounted from the images taken by a confocal microscope.

**Figure 6:**
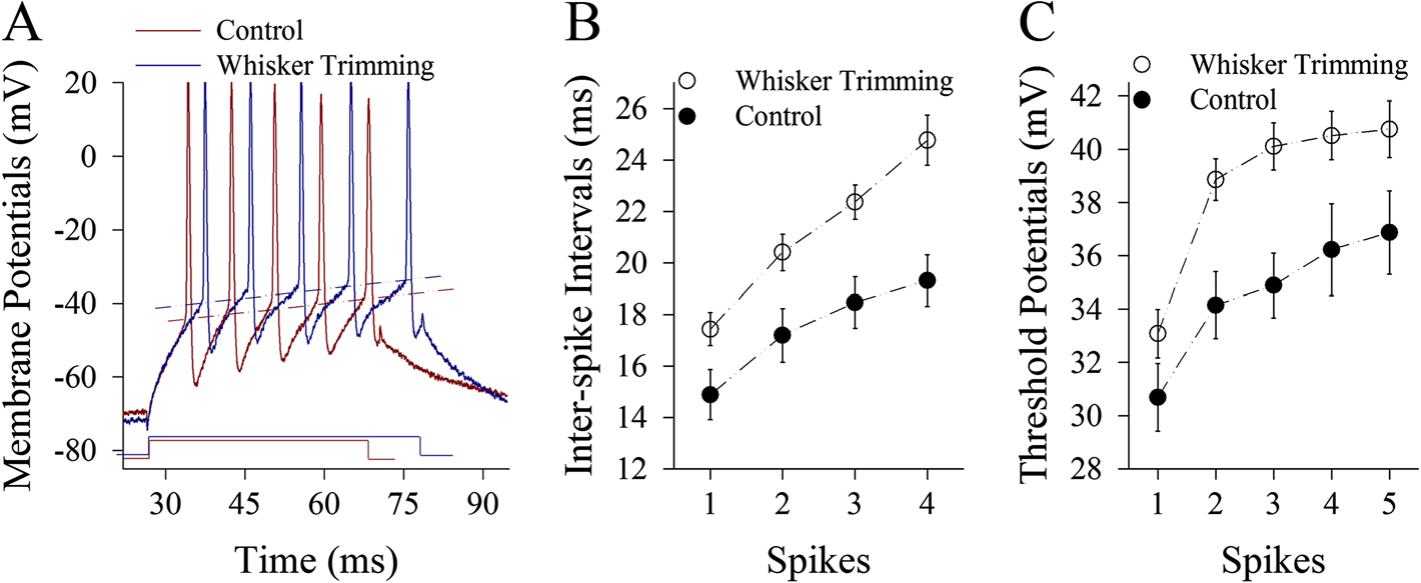
**The loss of whisker inputs downregulates the capability to fire action potentials in GABAergic neurons of the barrel cortices. **Sequential spikes were induced by the depolarization pulse in an intensity that was a threshold for inducing a spike by 10 ms of pulse for each neuron. **A) **shows sequential spikes induced at the GABAergic neurons from whisker-trimmed mouse (dark-blue trace) and control (dark-red trace). **B)** illustrates inter-spike intervals for spike 1~2 up to 4~5 in GABAergic neurons from whisker-trimmed mice (open symbols; n=15) and controls (filled; n=16, p<0.01). **C)** illustrates threshold potentials for spikes 1~5 in GABAergic neurons from whisker-trimmed mice (open symbols; n=15) and controls (filled; n=16, p<0.01).

Figure 6 illustrates the capability of converting excitatory input into digital spikes measured by inter-spike interval (ISI). These GABAergic neurons after loss of whisker inputs appear to have lower ability to encode spikes (dark-blue trace in Figure 6A), compared to the controls (dark-red). ISI values for spikes 1~2 up to 4~5 are 17.43±0.64, 20.41±0.7, 22.37±0.67 and 24.77±0.97 in the WT neurons (open symbols in Figure 6B, n=15); and 14.88±0.98, 17.2±1, 18.46±1.0 and 19.3±0.99 in the controls (filled, n=16). ISI values for corresponding spikes in two sources of neurons are statistically different (p<0.01). Therefore, the loss of whisker inputs attenuates the capability of GABAergic neurons to convert excitatory inputs intro the digital spikes.

Figure 6C illustrates VGSC-mediated threshold potentials (Vts) at GABAergic neurons. Vts values for spikes 1 up to 5 are 33.1±0.91, 38.86±0.78, 40.1±0.89, 40.52±0.91 and 40.75±1.1 in the WT neurons (open symbols; n=15), and are 30.69±1.27, 34.15±1.26, 34.88±1.22, 36.23±1.72 and 36.88±1.56 in the controls (filled, n=16). Vts values for corresponding spikes are statistically higher in the WT neurons than the controls (p<0.01). Thus, loss of whisker inputs attenuates the active intrinsic properties of inhibitory neurons in the barrel cortices.

**Figure 7:**
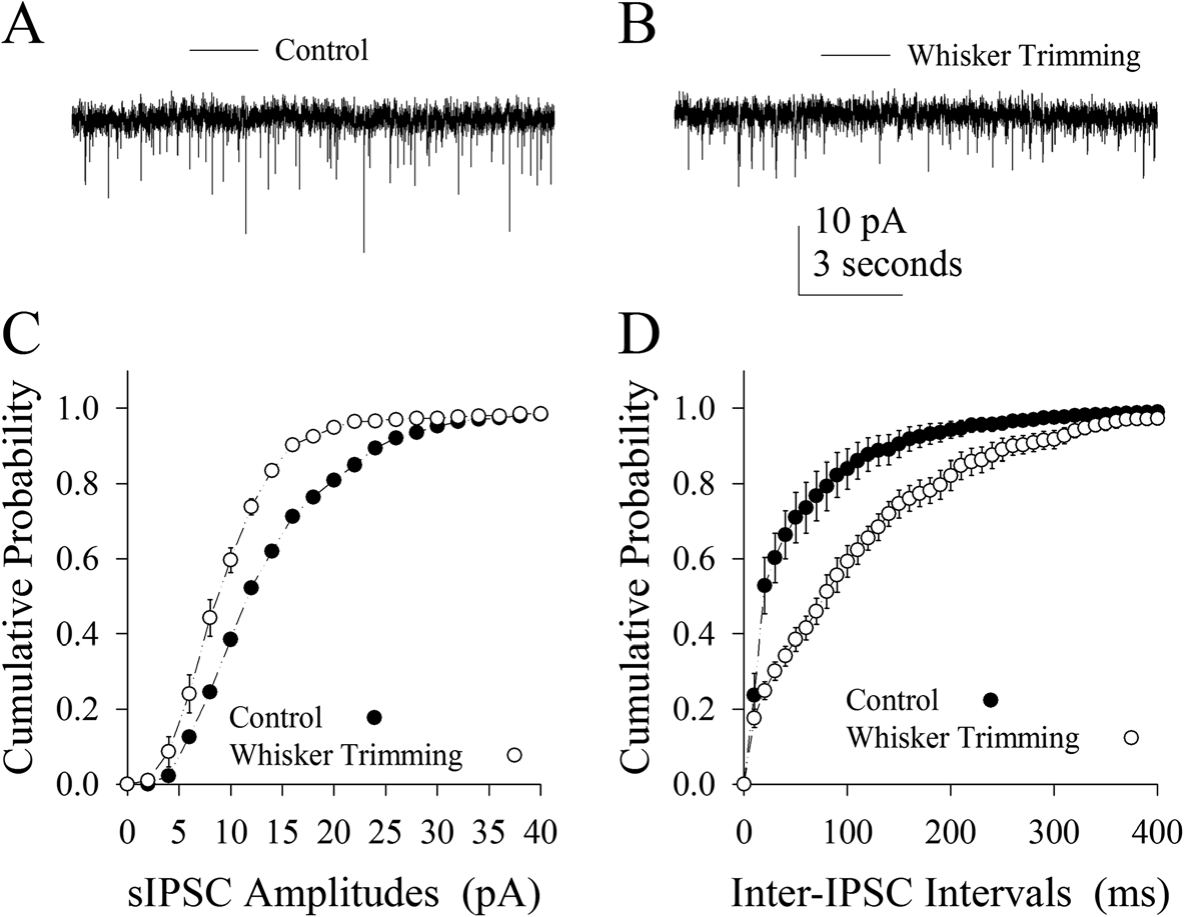
**The loss of whisker inputs downregulates the transmission of inhibitory synapses on pyramidal neurons of the barrel cortex. **The strength of inhibitory synapses was evaluated by recording spontaneous inhibitory postsynaptic currents (sIPSC) in the presence of 10 μM CNQX and 40 μM D-AP5, which were blocked by 10 μM bicuculine. **A) **shows sIPSCs recorded on a pyramidal neuron in the barrel cortex from a control mouse. **B) **shows sIPSCs recorded on a pyramidal neuron in the barrel cortex from a whisker-trimmed mouse. Calibration bars are 10 pA/3 seconds **C)** illustrates cumulative probability versus sIPSC amplitudes in barrel cortical pyramidal neurons from whisker-trimmed mice (open symbols, n=11) and controls (filled, n=12). **D) **illustrates cumulative probability versus inter-IPSC intervals in barrel cortical pyramidal neurons from whisker-trimmed mice (open symbols, n=11) and controls (filled, n=12).

Figure 7 illustrates the effects of loss of whisker inputs on inhibitory synaptic transmission. sIPSCs after whisker trimming appear to be lower (Figure 7B), compared to the controls (Figure 7A). Figure 7C shows cumulative probability vs. sIPSC amplitudes from the WT neurons (open symbols, n=11) and the controls (filled, n=12). Figure 7D shows cumulative probability vs. inter-sIPSC intervals from the WT neurons (open symbols, n=11) and the controls (filled, n=12). Statistical analysis indicates that sIPSC amplitudes and frequencies (1/inter-sIPSC interval) are lower in the WT neurons than in the controls. Therefore, loss of whisker inputs attenuates the inhibitory synaptic transmission including GABA release probability and receptor responsiveness in the barrel cortices.

**Figure 8:**
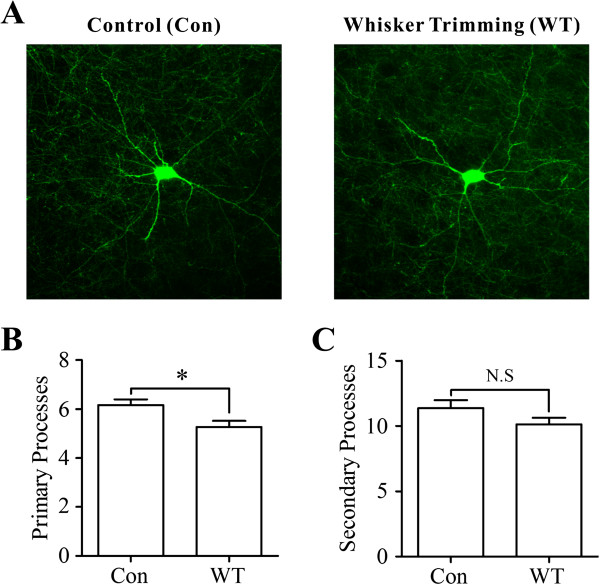
**A loss of whisker inputs leads to plastic changes in the processes of GABAergic neurons in the barrel cortices. A) **illustrates the images of GABAergic neurons and their processes from a control mouse (left panel) and a whisker-trimmed mouse (right). **B) **Statistical analysis illustrates primary processes on GABAergic neurons from whisker-trimmed mice and control (n=15, p<0.05). **C) **illustrates the secondary processes sprouted from the primary processes on GABAergic neurons from whisker-trimmed mice and controls (n=15, p=0.2).

In terms of the morphological changes in the barrel cortical GABAergic neurons, we analyzed the densities of processes, which reflected the volume of receiving inhibitory inputs. The number of processes appears to decrease on GABAergic neurons from the WT mice (right panel in Figure 8A), compared with those from the controls (left in 8A). Statistical analyses in Figure 8B-C illustrate the number of primary and secondary processes on somata from the WT mice and controls. Primary processes are 5.27±0.25 in the WT neurons and 6.12±0.23 in the controls (p<0.05, n=15). Secondary processes are 10.13±0.5 in the WT neurons and 11.39±0.59 in the controls (p=0.2). Therefore, the main processes for receiving presynaptic inputs decrease on GABAergic neurons after loss of whisker inputs.

## Discussion

In whisker-trimmed mice versus controls, we analyzed the changes of excitatory and inhibitory neurons in the barrel cortices. After loss of whisker inputs for a week, the functions of excitatory neurons and synapses as well as the sites of receiving excitatory inputs are upregulated (Figures 2 and 5). On the other hand, the functions of GABAergic neurons and synapses as well as the processes of receiving synaptic inputs are downregulated (Figures 6 and 8). These changes elevate the activity levels of network neurons in the barrel cortices, which may prevent a loss of their functions due to idle whisker inputs and increase their sensitivity to sensory inputs, as well as be ready to attracting the innervations from other sensory cortices and/or peripheral nerves for the remained modalities during the cross-modal sensory plasticity [39-42].

In terms of physiological impacts for bidirectional changes in pyramidal neurons vs. GABAergic neurons from the barrel cortex after the loss of whisker inputs, the upregulation of excitatory units and the downregulation of inhibitory units will reset the balance of excitation versus inhibition toward the end of excitation. In addition to reducing the threshold to boost neuronal networks, this upregulated activity may maintain the sensitivity of pyramidal neurons to weak input, so that their functions are not lost. Moreover, their upregulated activities may attract the exogenous inputs to innervate the barrel cortices, such as from piriform cortex [42], for cross-modal sensory plasticity and rehabilitation. Upregulations in the frequency of excitatory synaptic events (Figure 3) and the sites of receiving synaptic inputs (Figures 4 and 5) grant the establishment of new excitatory innervations in the barrel cortices.

After a loss of whisker inputs, the capabilities of firing spikes and transmitting excitatory synaptic signals increase on pyramidal neurons in the barrel cortex (Figures 2 and 3). The capabilities of firing spikes on GABAergic cells and executing their synaptic outputs decrease (Figures 6 and 7). That is, the intrinsic property and synaptic transmission change coordinately for homogenous functions in experience-dependent neural plasticity. This coordinate change is also seen synaptic transmission and input structures, since excitatory synaptic events and dendritic spines increase in a loss of whisker inputs (Figures 3 and 5). The coordination in the neurons and synapses is critical for them to work in a common purpose, i.e., the increase of neuronal sensitivity to inputs boost the activity of neuronal networks for cross-modal sensory plasticity [41,42]. In addition, we observed the bidirectional change between processes and their spines in apical and basal dendrites (Figures 4 and 5). This homeostasis in process density and spines saves the neuronal resources, a process similar to homeostasis by coordinating subcellular compartments and single molecules [43,44]. Therefore, the coordination and homeostasis among the neurons and synapses are present *in vivo*, based on our studies, which expends this knowledge obtained from the studies *in vitro*[45].

In terms of the mechanism underlying the upregulation of excitatory units and the downregulation of inhibitory units after loss of sensory inputs in the barrel cortices, we assume that they use homeostatic mechanisms, which are seen in the studies *in vitro*. Neuronal activities undergo homeostatic upregulation after functional deficits by pharmacological or genetic manipulations [45,46]. For instance, neuronal excitability rises when removing the treatment of TTX. The density of AMPA-type glutamate receptors is high when using CNQX. Neuronal excitability shows low and then recovery when potassium channels are over-expressed [47-49]. Such slowly developed homeostasis plays a role in functional compensation. The molecular mechanisms underlying neural homeostasis include glutamate/GABA receptors, voltage-gated sodium channels, brain-derived neurotrophic factors and α/β CaM-kinases [50-56]. It remains to be investigated how these molecules are coordinately initiated *in vivo* for the plasticity of the barrel cortices after loss of whisker inputs.

Excitatory synaptic transmission and dendritic spines increase on pyramidal neurons of the barrel cortex after loss of whisker inputs. As the strength of synaptic activities from the thalamus input deceases due to a lack of information from whisker-trigeminal ganglion-thalamus afferent pathway, these increased events and input-targeting units at excitatory synapses may be from cerebral cortices for other modalities, which is supported by our previous study [42]. This point brings insight into the concept that the neurons are never to be the functional silent units under the physiological condition. After loss of excitatory synaptic inputs, the neurons call up through the homeostatic mechanism, attract synaptic inputs from other cortical areas and execute new functions, e.g., cross-modal plasticity for sensory compensation. How the substitution of other cortical inputs to thalamus inputs is temporally controlled by the molecular events remains to be studied.

Previous studies in the barrel cortices after trimming whiskers indicated the changes in synaptic transmission [25,26], synaptic plasticity [27-30], dendritic reo rganization [31,32], spine generation [33,34] and zinc-containing neural circuit reorganization [35,36]. These data indicate the important roles of synaptic plasticity and circuit rewire in experience-dependent neural plasticity. By labeling different neurons as well as studying their functions and morphology, we are able to see the coordination and homeostasis among the different types of neurons and synapses in the barrel cortices after experience-dependent neural plasticity. Our study brings new information for this subject.

In summary, we have investigated experience-dependent plasticity in the barrel cortices after loss of whisker inputs. The upregulation of excitatory neurons and synapses as well as the downregulation of inhibitory neurons and synapses are associated with the loss of sensory inputs. The upregulated activities of network neurons, after loss of their original sensory inputs, will prevent the loss of their functions and attract the inputs from other cortical areas and/or peripheral nerves for cross-modal compensation.

## Methods and materials

The entire procedures were approved by Institutional Animal Care Unit Committee (IACUC) in the Administration Office of Laboratory Animals at Beijing China (B10831).

### A mouse model of removing whisker stimulus

In order to analyze the activities of barrel cortical neurons and synapses relevant to the changes in whiskers’ experience in cell-specific manner, we need the mice whose cortical neurons are labeled by different markers. We cross-matched the mice from strains of C57(Thy1YFP)BL/6N (from He in IBP-CAS) and FVB-Tg(GADGFP)4570Swn/J (Jackson Lab, USA). Pyramidal neurons in C57 mice were genetically labeled by yellow fluorescent protein (YFP), in which the promoter was Thy1 on the upstream of YFP. GABAergic neurons in FVB mice were labeled by green fluorescent protein (GFP), in which the promoter was GAD on the upstream of GFP. Such cross-matched mice possess YFP-labeled pyramidal neurons and GFP-labeled GABAergic neurons in cerebral cortices (Figure 1). The mice in postnatal days 7 were divided into two groups that were whisker trimming on right side and control (intact whiskers), respectively. The whisker trimming was given every day for one week with no trimming the furs in the face of mice. During the operation, the mice were placed in home-made cages, in which their running and motion were restricted, but the extensions of their bodies and arms were allowed. The cares were taken including no stress and circadian disturbance to the mice. In addition, the mice with normal whisking and symmetric whiskers were selected for our experiments.

### Brain slices and neurons

The cortical slices (400 μm) were prepared from the mice with whisker trimming and control. They were anesthetized by inhaling isoflurane and decapitated by guillotine. Slices were cut with a Vibratome in oxygenated (95% O_2_ and 5% CO_2_) artificial cerebrospinal fluid (ACSF), in which the concentrations (mM) of different elements were 124 NaCl, 3 KCl, 1.2 NaH_2_PO_4_, 26 NaHCO_3_, 0.5 CaCl_2_, 4 MgSO_4_, 10 dextrose, and 5 HEPES, pH 7.35 at 4°C. The slices were held in the oxygenated ACSF (124 NaCl, 3 KCl, 1.2 NaH_2_PO_4_, 26 NaHCO_3_, 2.4 CaCl_2_, 1.3 MgSO_4_, 10 dextrose, and 5 HEPES, pH 7.35) at 25°C for 2 hours. A slice was transferred to a submersion chamber (Warner RC-26G) that was perfused with the ACSF oxygenated at 31°C for whole-cell recording [41,42,57-60]. Chemical reagents were from Sigma.

The neurons in the barrel cortical slices are showed GFP-labeling for GABAergic cells and YFP-labeling for pyramidal cells. These neurons in layers II-III were selected for whole-cell recordings under DIC-fluorescent microscope (Nikon FN-E600, Japan), in which the excitation wavelength was 488 nm. GABAergic neurons showed fast spiking without the adaptation in spike amplitude and frequency, typical properties for interneurons [41,61-64]. Cortical pyramidal neurons demonstrated regular spikes with the adaptation in their amplitudes and frequency.

### Whole-cell recording and neuronal functions

Cortical neurons were recorded by an MultiClamp-700B amplifier under voltage-clamp for their synaptic activity and current-clamp for their active intrinsic properties. The electrical signals were inputted into pClamp-10 (Axon Instrument Inc, USA) for the data acquisition and analysis. The output bandwidth in this amplifier was 3 kHz. Pipette solution for studying excitatory events included (mM) 150 K-gluconate, 5 NaCl, 5 HEPES, 0.4 EGTA, 4 Mg-ATP, 0.5 Tris-GTP, and 5 phosphocreatine (pH 7.35; [65]. The solution to record inhibitory synapses contained (mM) 130 K-gluconate, 20 KCl, 5 NaCl, 5 HEPES, 0.5 EGTA, 4 Mg-ATP, 0.5 Tris–GTP and 5 phosphocreatine [66]. These pipette solutions were freshly made and filtered (0.1 μm). The osmolarity was 295~305 mOsmol and pipette resistance was 5~6 MΩ.

The functions of GABAergic neurons were assessed based on their active intrinsic properties and inhibitory outputs [67]. The functional status of their inhibitory outputs were evaluated by recording spontaneous IPSCs (sIPSC) under voltage-clamp on pyramidal neurons in the presence of 10 μM 6-Cyano-7-nitroquinoxaline-2,3-(1H,4H)-dione (CNQX) and 40 μM D-amino-5-phosphonovanolenic acid (D-AP5) in ACSF to block ionotropic glutamate receptors and to isolate IPSCs [66]. 10 μM bicuculline was washed into the slices at the end of experiments to test whether synaptic responses were mediated by GABA_A_R, which did block sIPSCs in our experiments. The series and input resistances for all of the neurons were monitored by injecting hyperpolarization pulses (5 mV/50 ms), and calculated by voltage pulses versus instantaneous and steady-state currents. It is noteworthy that the pipette solution with the high concentration of chloride ions makes the reversal potential to be −42 mV. sIPSCs are inward when the membrane holding potential at −65 mV [66].

The functions of pyramidal neurons were assessed based on their active intrinsic properties and excitatory outputs [67]. The functional status of their excitatory outputs were evaluated by recording spontaneous EPSCs (sEPSC) under voltage-clamp on cortical pyramidal cells in presence of 10 μM bicuculline in ACSF to block ionotropic GABA receptors and isolate EPSCs. 10 μM CNQX and 40 μM DAP-5 were added into ACSF perfused into the slices at the end of experiments to test whether synaptic responses were mediated by GluR, which did block sEPSCs in our study. In addition, series and input resistances for all of these neurons were monitored by injecting hyperpolarization pulses (5 mV/50 ms), and calculated by voltage pulses vs. instantaneous and steady-state currents.

Action potentials at these cortical neurons were induced by injecting depolarization pulses, whose intensity and duration were changed based on the aim of experiments. The ability to convert excitatory inputs into sequential spikes was evaluated by inter-spike intervals (ISI) when depolarization pulses (200 ms in the duration and threshold for 10 ms pulse-induced spike for the intensity) were given [68]. Neuronal intrinsic properties in our study included spike threshold potential (Vts) and absolute refractory period (ARP). Vts were the voltages of spike-onsets [43,63,69-71].

Data were analyzed if the recorded neurons had the resting membrane potentials negatively more than −60 mV, and action potential amplitudes more than 90 mV. The criteria for the acceptance of each experiment also included less than 5% changes in resting membrane potential, spike magnitude, and input resistance throughout each experiment. Input resistance was monitored by measuring cellular responses to hyperpolarization pulse at the same values as the depolarization that evoked action potentials. To estimate the effect of whisker trimming on neuronal spikes and synaptic transmission, we measured sEPSC, sIPSC ISI and Vts in the neurons from mice of control and whisker trimming. The differences in sEPSC, sIPSC, ISI and Vts were presented as mean±SE. The comparisons of these data were done by t-test.

### The morphological studies of GABAergic neurons and pyramidal neuons in barrel cortices

The mice in one week after whisker trimming and controls were anesthetized by the intraperitoneal injection of sodium pentobarbital, and were perfused by 4% paraformaldehyde in 0.1 M phosphate buffer solution (PBS) from left ventricle/aorta until the body was rigid. The brains were quickly isolated and fixed in 4% paraformaldehyde PBS for additional 24 hours. Cortical tissues were sliced in the cross section of barrel cortex at 60 μm by a Vibratome. Sections were washed by PBS for 3 times, air-dried and cover-slipped. The images in the structures of YFP-labeled pyramidal neurons and GFP-GABAergic cells in the cross-sections of barrel cortices were photographed under a laser scanning confocal microscopy (Olympus FV-1000, Japan), in which their fluorescent markers were deconvoluted by 510 nm and 540 nm [72].

The structures of these neurons were analyzed by a commercialized software MetaMorph in Meta Imaging Series (ver. 6.1, Universal Imaging Cooperation in Molecular Device). As the brain tissues were sliced in series sections, the counting and analysis in cell structures were able to be done at least from two sections for each of barrels. The analyzed sections were chosen in a manner of one section from every two in order to prevent the influence of cells that crossed the neighboring sections on the analysis. In the analyses of dendrites, the primary processes (branches from somata) and secondary ones (branches from primaries) of pyramidal and GABAergic neurons were measured in each of barrel sections. In pyramidal neurons, the analyses of their dendrites included the apical and basal dendrites [41]. The spines were the protrusion extended from on the dendrites, which were accounted as spines per 10 μm.

## Competing interests

The authors declare that they have no competing interests.

## Authors’ contributions

GZ and ZG carried out the experiments and data analyses. JHW contributed to project design and paper writing. All authors read and approved the final manuscript.
